# Systematic Assessment of the Impact of DTI Methodology on Fractional Anisotropy Measures in Alzheimer’s Disease

**DOI:** 10.3390/tomography7010003

**Published:** 2021-02-06

**Authors:** Maurizio Bergamino, Elizabeth G. Keeling, Ryan R. Walsh, Ashley M. Stokes

**Affiliations:** 1 Division of Neuroimaging Research, Barrow Neurological Institute, Phoenix, AZ 85013, USA; maurizio.bergamino@Barrowneuro.org (M.B.); ekeeling@asu.edu (E.G.K.); 2 School of Life Sciences, Arizona State University, Tempe, AZ 85013, USA; 3 Muhammad Ali Parkinson Center, Barrow Neurological Institute, Phoenix, AZ 85013, USA; ryan.walsh@Barrowneuro.org

**Keywords:** Alzheimer’s disease, mild cognitive impairment, diffusion tensor MRI, cognitive decline, fitting algorithms

## Abstract

White matter microstructural changes in Alzheimer’s disease (AD) are often assessed using fractional anisotropy (FA) obtained from diffusion tensor imaging (DTI). FA depends on the acquisition and analysis methods, including the fitting algorithm. In this study, we compared FA maps from different acquisitions and fitting algorithms in AD, mild cognitive impairment (MCI), and healthy controls (HCs) using the Alzheimer’s Disease Neuroimaging Initiative (ADNI) database. Three acquisitions from two vendors were compared (Siemens 30, GE 48, and Siemens 54 directions). DTI data were fit using nine fitting algorithms (four linear least squares (LLS), two weighted LLS (WLLS), and three non-linear LLS (NLLS) from four software tools (FSL, DSI-Studio, CAMINO, and AFNI). Different cluster volumes and effect-sizes were observed across acquisitions and fits, but higher consistency was observed as the number of diffusion directions increased. Significant differences were observed between HC and AD groups for all acquisitions, while significant differences between HC and MCI groups were only observed for GE48 and SI54. Using the intraclass correlation coefficient, AFNI–LLS and CAMINO–RESTORE were the least consistent with the other algorithms. By combining data across all three acquisitions and nine fits, differences between AD and HC/MCI groups were observed in the fornix and corpus callosum, indicating FA differences in these regions may be robust DTI-based biomarkers. This study demonstrates that comparisons of FA across aging populations could be confounded by variability in acquisitions and fit methodologies and that identifying the most robust DTI methodology is critical to provide more reliable DTI-based neuroimaging biomarkers for assessing microstructural changes in AD.

## 1. Introduction

Dementia is characterized clinically by a gradual decline in multiple cognitive domains, including language, memory, executive, and visuospatial functions, which ultimately lead to an inability to perform instrumental and/or basic activities of daily living and ultimately death. Alzheimer’s disease (AD) accounts for up to 80% of all dementia diagnoses [1] and is characterized pathologically by extracellular amyloid plaques, intraneuronal neurofibrillary tangles, and neurodegeneration [2]. Mild cognitive impairment (MCI) usually precedes Alzheimer’s disease [3], and individuals with MCI have an increased risk of dementia with age [4]. Imaging biomarkers for AD and MCI include both positron emission tomography (PET) (e.g., using amyloid and tau tracers) and magnetic resonance imaging (MRI) methods, the latter of which are used to assess structural, microstructural, and other pathophysiological characteristics of AD [5].

MR-based diffusion tensor imaging (DTI) reports on microstructural properties of white matter (WM), and the derived metrics have been applied extensively as neuroimaging biomarkers to study a range of clinical conditions [6], including AD and MCI [7]. DTI-derived metrics, such as fractional anisotropy (FA) and axial and radial diffusivities (AxD and RxD, respectively), are indicative of water diffusion around WM tracts, which is radially restricted by the myelin sheath surrounding axons. In the context of MCI and AD, DTI metrics have demonstrated microstructural abnormalities in several WM areas, including the cingulum, fornix, corpus callosum (CC), and uncinate fasciculus (UF), in addition to temporal, occipital, and frontal WM [8,9,10]. Cognitive scores, including those relating to memory and executive function, have been found to correlate with DTI-derived metrics [11], suggesting a microstructural component to cognitive changes. Additionally, DTI has been shown to be sensitive to WM degeneration in the early stages of AD, including MCI [12]. It is important to note, however, that pathologically proven direct correlations between WM changes and DTI metrics remain elusive.

Although DTI is widely used, the results can be sensitive to data acquisition, pre-processing, and analysis. With recent improvements in scanner hardware and image acceleration, DTI acquisitions now typically include a large number of DTI directions (>30) and/or multiple shells (as a function of b-value). Permutations of DTI analysis include various image coregistration/normalization procedures [13], and the choice of regional analysis, voxel-based analysis, or skeletonized analysis (also called tract-based spatial statistics (TBSS)) [14]. Methodological differences can lead to varying results, even within the same cohort of subjects [13]. This is true across neurodegenerative disease states, including Parkinson’s disease [15].

Another important factor in DTI analysis is the fitting procedure used to estimate the diffusion tensor [13]. Linear least squares (LLS) regression is the most basic model used in diffusion MRI for the estimation of diffusion parameters. Higher accuracy can be obtained by using weighted linear least squares (WLLS) regression, but this method is slower than LLS. Non-linear least squares regression (NLLS) and other more robust estimators, such as robust estimation of tensors by outlier rejection (RESTORE) [16] or informed RESTORE (iRESTORE) [17] methods, can also be implemented. While software tools are freely available to perform these model fits, to the best of our knowledge no study has systematically investigated the impact of differing fitting methodologies on the resulting WM integrity metrics in AD and MCI subjects. Indeed, over-fitting is a common error likely present in many reported studies that are not either internally or externally validated with independent cohort/fitting analyses.

In this study, we examined group-level differences in WM integrity, as quantified by FA, across AD, MCI, and healthy control (HC) subjects obtained from the Alzheimer’s Disease Neuroimaging Initiative (ADNI) database (https://ida.loni.usc.edu/ accessed on 5 February 2021). Three different DTI acquisitions from two scanner vendors (Siemens 30, General Electric (GE) 48, and Siemens 54 directions) were compared using nine commonly utilized fitting procedures with voxel-based analysis (VBA). Correlations between FA and cognitive scores, including the Montreal Cognitive Assessment (MoCA) [18], the Mini-Mental State Examination (MMSE) [19], and the Alzheimer’s Disease Assessment Scale (ADAS) [20] were also assessed. The intraclass correlation coefficient (ICC) [21] was used to evaluate the consistency across fitting algorithms. The aim of this study is to evaluate the impact of different dMRI acquisitions and DTI fitting procedures on FA measures in the context of AD. These findings may be relevant in the interpretation of microstructural changes associated with Alzheimer’s pathology, and more broadly in other neurodegenerative pathologies.

## 2. Methodology

### 2.1. Subjects

Data from HC, MCI, and AD subjects were downloaded from the ADNI-3 database. Single-shell DTI data were available with three different acquisitions (30, 48, and 54 directions) from two MRI vendors (Siemens and GE). Across all acquisitions and vendors, a total of 159 HC, 79 MCI, and 30 AD subjects were included (see Table 1 for the complete breakdown of each cohort). The Siemens 30 (SI30) cohort included 68 subjects, comprised of 38 HC, 23 MCI, and 7 AD. The GE 48 (GE48) cohort included 73 subjects, comprised of 41 HC, 20 MCI, and 12 AD. The Siemens 54 (SI54) cohort included 127 subjects, comprised of 80 HC, 36 MCI, and 11 AD. MoCA, MMSE, and ADAS scores were obtained from ADNI for correlations with DTI–FA. The MoCA and MMSE are standard multi-domain cognitive assessments, with the MoCA focusing more on earlier detection of MCI [18,19]. The ADAS is the standard for assessing the level of cognitive dysfunction in Alzheimer’s disease [20]. Higher scores on the MoCA and MMSE indicate greater cognitive function, while higher scores on the ADAS indicate greater cognitive impairment. All subject characteristics and scores are summarized in Table 1.

### 2.2. MRI Protocols

All data were acquired at 3T using single-shell DTI acquisitions. For SI30, a Siemens Biograph mMR scanner (Siemens Healthcare, Erlangen, Germany) was used with the following parameters: 30 diffusion-encoding directions (*b*-value: 1000 s/mm^2^; TR/TE: 12400/95.0 ms; flip-angle = 90°; matrix: 116 × 116; voxel size 2.0 mm × 2.0 mm; slice thickness: 2.0 mm; number of averages = 1) and one B0 image at the beginning of the acquisition. For GE48, a GE Discovery MR750 scanner (GE Healthcare, Waukesha, WI, USA) was used with the following parameters: 48 diffusion-encoding directions (*b*-value: 1000 s/mm^2^; TR/TE: 7800/60.4 ms; flip-angle = 90°; matrix: 256 × 256; voxel size 0.906 mm × 0.906 mm; slice thickness: 2.0 mm; number of averages = 1) and six non-diffusion-weighted images (B0 images) at the beginning of the acquisition. For SI54, a Siemens MAGNETOM Prisma scanner (Siemens Healthcare, Erlangen, Germany) was used with the following parameters: 54 diffusion-encoding directions (*b*-value: 1000 s/mm^2^; TR/TE: 7200/56.0 ms; flip-angle = 90°; matrix: 116 × 116; voxel size 2.0 mm × 2.0 mm; slice thickness: 2.0 mm; number of averages = 1) and seven B0 images.

### 2.3. DTI Processing and Fittings

All DTI DICOM images were converted to NIFTI using *dcm2niix* and were identically preprocessed using the functional magnetic resonance imaging of the brain (FMRIB) software library tool (FSL, version 6.0.0, Oxford, UK) [22]. The raw DTI images were corrected for motion and eddy currents by *eddy* (FSL) [23]. To account for the rotational component of registration, the b-vector files were compensated after motion correction and prior to calculating the b matrices. A brain mask was defined for each subject on the averaged B0 images using the brain extraction toolbox (*bet*) [24]. Using the B0 images from all subjects (n = 268), a group template was created using *antsMultivariateTemplateConstruction.sh* included in the advanced normalization tools (ANTs) (http://stnava.github.io/ANTs/ accessed on 5 February 2021). This group template was used as the standard space for subsequent analyses. FA maps, created by the different fitting algorithms described below, were coregistered to this template through ANTs with a symmetric image normalization (SyN) algorithm [25]. All FA maps in template space were smoothed using FSL with an isotropic Gaussian kernel (sigma, 3 mm).

For each acquisition, nine fitting algorithms were used to estimate the diffusion tensor and to calculate FA maps: unweighted linear (LLS: FSL, DSI Studio, CAMINO, and the Analysis of Functional NeuroImages (AFNI)); weighted linear (WLLS: FSL and CAMINO); non-linear least squares (NLLS: CAMINO and AFNI); and iteratively reweighted non-linear least squares (RESTORE: CAMINO) regressions. All fitting algorithms and inputs used are shown in Table 2.

### 2.4. Statistical Analysis

Age, MMSE, MoCA, and ADAS scores are presented as mean and standard deviation (SD) for each group and acquisition. Differences in age and cognitive test scores across groups and acquisitions were assessed using the Kruskal–Wallis rank-sum test. For significant *p*-values (*p* < 0.05), post hoc comparisons were assessed using the Dunn’s test with Holm–Sidak adjustment (Table 1).

As unequal sample sizes using ANCOVA may increase type I error, particularly with heterogeneous variance across the groups [26], Bartlett’s test of equal variance was first performed across all groups using GE48 and across all scanners using the HC group. All analyses on the FA maps were performed at the voxel-based level using analysis of covariance (VBA–ANCOVA), with age and gender as covariates. For each acquisition and FA map, VBA–ANCOVA effect-sizes through the partial eta-squared (*η*^2^_-p_) index were used to explore differences across groups (AD vs. MCI vs. HC), which enables quantitative comparisons across studies [27,28]. Significant clusters are reported at *η*^2^_-p_ > 0.15 (large effect) and size > 100 voxels. Subsequent VBA post hoc comparisons across groups were assessed using the effect-size through the Hedges’ g (*g*) index. Significant clusters are reported at |*g*| > 0.85 (large effect) and size > 100 voxels [23]. In addition, the individual results obtained for each DTI acquisition and each fit were averaged together to have a more robust group-wise comparison, as previously shown [29].

The VBA correlations between FA maps and cognitive test scores were assessed using Spearman’s correlation coefficient (*r*), with age and gender as covariates. Significant clusters are reported at |*r*| > 0.50 and size > 100 voxels. VBA effect-size analysis and correlations were only performed within WM, as defined using a FA threshold of 0.20. 

For ICC analysis, a two-way mixed model was used, treating the nine fitting procedures as fixed effects in one factor and subjects as random-effects in the other factor (ICC (3, 1)). VBA–ANCOVA and post hoc comparisons across groups were performed using R (version 3.5.1, R Foundation for Statistical Computing, Vienna, Austria. URL https://www.R-project.org/ accessed on 5 February 2021) and RStudio (version 1.0.143, RStudio: Integrated Development for R. RStudio, PBC, Boston, MA, USA. http://www.rstudio.com/ accessed on 5 February 2021), along with the RNifti package (version 0.8.1, https://github.com/jonclayden/RNifti accessed on 5 February 2021) to load DTI images into R. The ICC across fits for each acquisition and the Spearman’s correlations were performed using in-house Matlab scripts (Matlab version R2019a, The MathWorks Inc., Natick, MA, USA).

The clusters identified as statistically different across groups were labeled according to the JHU white-matter tractography and the ICBM-DTI-81 white-matter label atlases [30,31]. The abbreviations of the WM locations from these atlases, used in all figures, are summarized in Supplementary Table S1.

## 3. Results 

### 3.1. Subjects

The Kruskal–Wallis rank sum test showed significant differences for MMSE, MoCA, and ADAS score across the three groups (*p* < 0.001) for each acquisition; post hoc analysis identified differences for all scores across all groups, except for ADAS between AD and MCI for the GE48 acquisition (Z = 1.93, *p* = 0.054). No differences in age were found for any acquisition (SI30: H = 5.8, *p* = 0.054; GE48: H = 4.7, *p* = 0.094; SI54: H = 6.1, *p* = 0.051) (Table 1). Comparing all acquisitions, significant differences were observed for MoCA scores across AD groups between SI30 and SI54 (H = 6.90, *p* = 0.03; Dunn’s test). No differences in age were found for all groups (HC: H = 1.50, *p* = 0.052; MCI: H = 0.21, *p* = 0.900; AD: H = 1.14, *p* = 0.565) (Table 1).

### 3.2. ANCOVA Results: FSL–LLS

**Figure 1:**
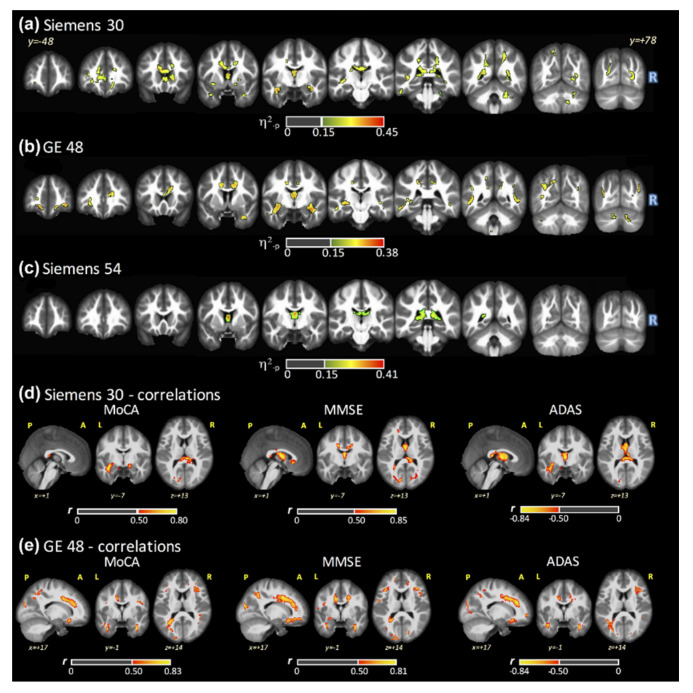
Significant clusters (*η*^2^_-p_ > 0.15 and size > 100 voxels) obtained with ANCOVA for fractional anisotropy (FA) from FSL (linear least square (LLS) algorithm) for (**a**) SI30, (**b**) GE48, and (**c**) SI54. Significant Spearman’s correlations (|*r*| > 0.50 and size > 100 voxels) from FSL (LLS algorithm) between the cognitive tests and FA maps from (**d**) SI30 and (**e**) GE48. No significant correlations were found for SI54.

Bartlett’s test of equal variance revealed no differences in variance across groups using the GE48 acquisition (*p* = 0.895) or across scanners using the HC cohort (*p* = 0.608). Maps of *η*^2^_-p_ for FA generated by a single fit (FSL–LLS regression) are shown in Figure 1 across the three DTI acquisitions (panels a–c). Significant group-wise differences were found for all acquisitions across subject groups (AD, MCI, and HC), with significant clusters in the anterior thalamic radiation (ATR), CC, and fornix. The latter represents the largest WM cluster of group differences across acquisitions (covering 87%, 67%, and 90% of the fornix with SI30, GE48, and SI54, respectively). Statistically significant clusters in the cingulum of the cingulate gyrus (CCG) and forceps minor were also observed with both SI30 and GE48, while a significant cluster in the forceps major was found with both SI30 and SI54. For FSL–LLS, the post hoc comparisons between groups (HC versus AD, MCI versus AD, and HC versus MCI) are shown in the first column of Supplementary Figures S1–S3, respectively. These results are described in more detail below across all fitting algorithms.

### 3.3. FA Maps and Cognitive Scores Correlations

Figure 1 panels (d) and (e) show the significant clusters from VBA Spearman’s correlations using FSL–LLS between the cognitive tests and FA maps for SI30 (panel d) and GE48 (panel e). No significant correlations were found for SI54. Supplementary Figure S4 shows the comparison of the Spearman’s *r* index across all fitting algorithms. Positive correlations were found for MoCA and MMSE and negative correlations were found for ADAS in several WM locations for both SI30 and GE48. However, some negative correlations for MoCA and MMSE and positive correlations for ADAS were also detected for GE48. Differences in the Spearman’s *r* index, across the fitting procedures, were identified for both acquisitions.

### 3.4. ANCOVA Results: All Fits

**Figure 2:**
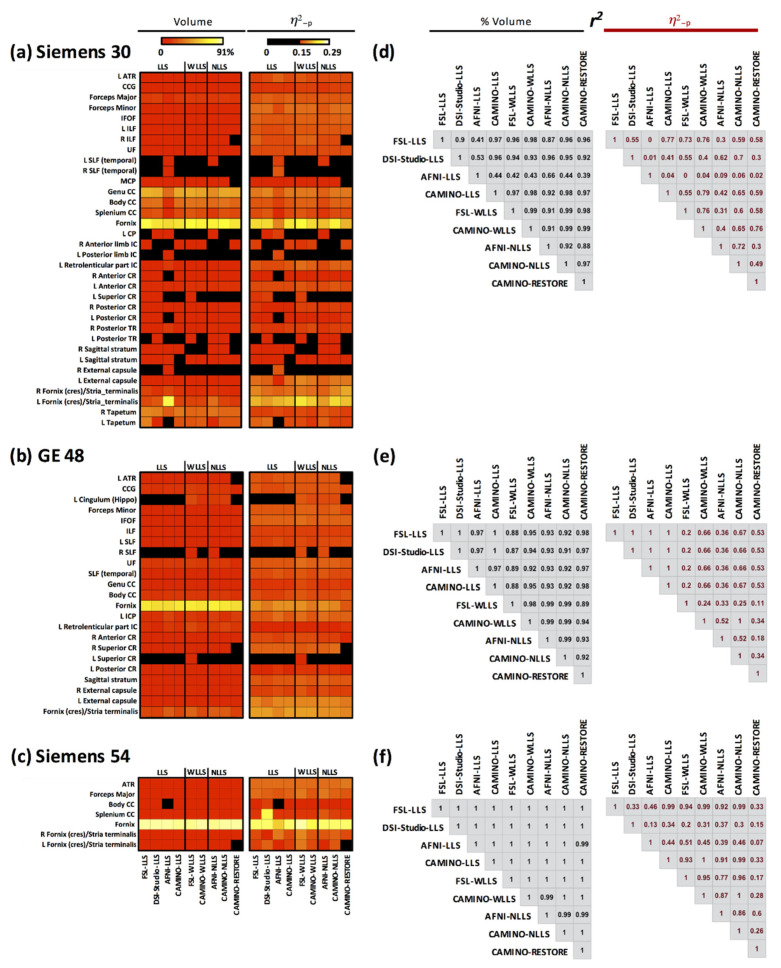
ANCOVA comparisons across all fitting algorithms for cluster volume and *η*^2^_-p_ with the relative pair-wise linear correlations for (**a**) SI30, (**b**) GE48, and (**c**) SI54. Significant clusters with *η*^2^_-p_ > 0.15 and size > 100 voxels. Panels (**d**–**f**) shows the correlation matrices between fits for each acquisition.

Figure 2 shows the volumes and *η*^2^_-p_ for the significant clusters of group-level differences calculated using the ANCOVA effect-size for each acquisition (Panels a–c: SI30, GE48, and SI54). For FSL–LLS shown in Figure 1, the regional differences from ANCOVA are shown in the first column of Figure 2, while the remaining columns show the results from the remaining fitting algorithms. Most individual regions were found using SI30, while the fewest regions were found using SI54. Within each acquisition, regional differences are also seen across the fitting algorithms, even for the same regression type. In some cases, regional differences were only observed with one or two methods; for instance, group differences in the right superior longitudinal fasciculus (SLF) were detected only by FSL–WLLS and AFNI–NLLS with GE48. For GE48 and SI54, all LLS methods provided similar clusters within each acquisition, except the body of the CC for SI54 using AFNI–LLS. With only 30 directions, LLS fits were more variable across many regions. Both WLLS and NLLS methods produced similar results, particularly for GE48 and SI54. Of all regions, the fornix yielded the highest volume clusters across all acquisitions and fit methods.

The correlation matrix (Panels d–f, Figure 2) for the cluster volume displays *r*^2^ values greater than ≥0.87 for GE48 (panel e), indicating good volumetric agreement across all fitting algorithms. On the other hand, for *η*^2^_-p_, all WLLS (FSL–WLLS and CAMINO–WLLS) and NLLS (AFNI–NLLS, CAMINO–NLLS, and CAMINO–RESTORE) exhibit small *r*^2^ values, suggesting poor agreement for effect size compared with the other fitting methods. Lower correlation values are observed for SI30, particularly for AFNI–LLS (*r*^2^ ≤ 0.66 and *r*^2^ ≤ 0.09 for the cluster volumes and *η*^2^_-p_ index, respectively), indicative of poor agreement compared with the other fitting methods. With more directions, fit consistency is much higher for SI54 (*r*^2^ ≥ 0.99 for cluster volume), indicating excellent agreement across all fitting algorithms. However, less consistency is observed for *η*^2^_-p_ index, indicating that volume differences are consistent across fits, but the effect sizes are less reliable. In other words, volumetric differences are less sensitive to the fit type when more directions are used, but the magnitude of the difference (i.e., effect size) remains sensitive to the type of fit.

### 3.5. Post Hoc: AD versus HC

**Figure 3:**
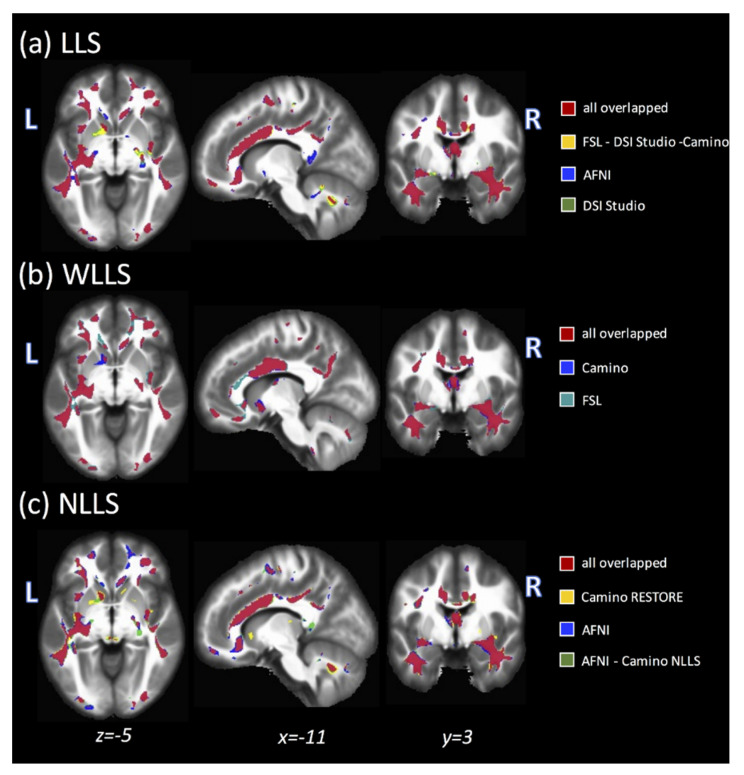
Overlap maps created from each fit method ((**a**) LLS, (**b**) weighted LLS (WLLS), and (**c**) non-linear LLS (NLLS)) for FA clusters where significant differences were found between AD and healthy control (HC) acquired using GE48. Color-coded voxels indicate regions identified with one or more fits, where red indicates regions where all fits overlapped (four for LLS, two for WLLS, and three for NLLS).

Figure 3 shows the overlapping significant clusters across fit algorithms for GE48 (FA: AD versus HC), separated by LLS (**a**), WLLS (**b**), and NLLS (**c**). The clusters in red indicate overlap across all fits (four for LLS, two for WLLS, and three for NLLS), and all other colors indicate clusters obtained with a subset of all fits. There is generally strong regional agreement across fit types and software tools. Subtle differences can be seen for AFNI–LLS (blue) relative to the other LLS fits (yellow). Additionally, both WLLS algorithms (**b**) produced smaller clusters in the CC and cerebellum compared to LLS (**a**) and NLLS (**a**).

Supplementary Figure S1 shows the post hoc comparisons between AD and HC groups for all acquisitions. Lower FA values were found in AD, compared with HC, in the majority of WM locations; however, some small clusters where FA in AD was higher than HC were also observed. In most cases, the regional differences were consistent within a fitting algorithm. A few exceptions include the left medial lemniscus for SI30; the inferior cerebellar peduncle (ICP), the left cerebral peduncle (CP), and left tapetum for GE48; and the right retrolenticular part of IC for SI54, which were found only by AFNI (LLS and/or NLLS). For all acquisitions, the largest cluster where decreased FA values in AD were found was the fornix (mean cluster volume ≈ 77% and mean *g* ≈ −1.49 for SI30; mean cluster volume ≈ 75% and mean *g* ≈ −1.21 for GE48; mean cluster volume ≈ 94% and mean *g* ≈ −1.58 for SI54). All acquisitions and fits also found lower FA values in AD in the CC, the forceps major and minor, and the CCG. On the other hand, clusters with lower FA values in HC, compared with AD, were detected in the left cortical spinal tract (CST, left ICP, right posterior limb of IC, right anterior limb of IC, left superior and posterior CR. The correlation matrices (d–f) show low *r*^2^ values for AFNI fits for the *g* index for all acquisitions. Consistent with ANCOVA, higher *r*^2^ values were observed for acquisitions with more directions and for volumes compared to effect sizes.

### 3.6. Post Hoc: AD versus MCI

Supplementary Figure S2 shows the post hoc comparisons between AD and MCI groups for all acquisitions. Differences between these two groups were found by SI30 and GE48, though no differences were detected for SI54. Lower FA values were found in AD compared with the MCI in several WM areas, such as CC, fornix, and CCG. However, we found significant clusters with lower FA values in MCI compared with AD in the ATR, CST, and anterior and posterior limb of IC for SI30 and the left ICP and left anterior limb of IC for GE48. Additionally, the correlation matrices show differences across the fitting algorithms, where AFNI–LLS methods produced lower *r*^2^ values compared with the other algorithms.

### 3.7. Post Hoc: MCI versus HC

Supplementary Figure S3 shows the post hoc comparisons between HC and MCI groups, where differences in FA values between HC and MCI groups were only found using GE48 and SI54 protocols. GE48 detected lower FA values in MCI compared with HC in a small cluster (volume < 4%) in the left CCG with FSL (both LLS and WLLS), DSI-Studio–LLS, and CAMINO–LLS. For SI54, all fits detected lower FA values in MCI compared with HC in the splenium of CC (volume < 8%) and fornix (volume ≈ 76%).

### 3.8. Intraclass Correlation Coefficient across Fits

**Figure 4:**
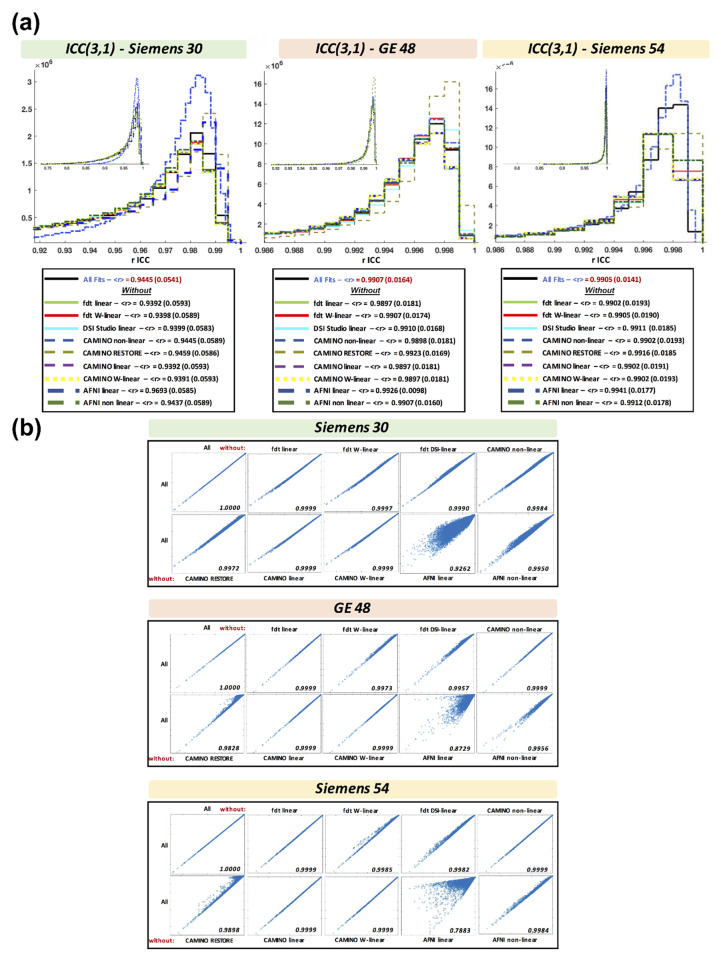
(**a**) Intraclass correlation coefficient (ICC) (3,1) across fit procedures for Siemens 30, GE 48, and Siemens 54. (**b**) Correlations for the *r*-ICC between all-fits and all-fits less one (leave-one-out methodology). CAMINO–RESTORE and AFNI–LLS have less similarity compared with the other fitting algorithms.

Figure 4 panel (**a**) shows the ICC results across acquisitions and fits. The *r*-ICC shows excellent similarity between fits for all acquisitions (*r*-ICC > 0.90) [32]. However, compared with GE48 and SI54, the SI30 acquisition showed a lower *r*-ICC across all fits (mean *r*-ICC_SI30_ = 0.94 versus *r*-ICC_GE48_ = 0.99 and *r*-ICC_SI54_ = 0.99). The *r*-ICC was slightly increased when CAMINO–RESTORE (∆*r*-ICC_GE48_ = +0.0016, ∆*r*-ICC_SI30_ = +0.0014, ∆*r*-ICC_SI54_ = +0.0011) and AFNI–LLS (∆*r*-ICC_GE48_ = +0.0020, ∆*r*-ICC_SI30_ = +0.0248, ∆*r*-ICC_SI54_ = +0.0036) were removed from analysis. This indicates that most of the algorithms produce highly similar results, while CAMINO–RESTORE and AFNI–LLS are less similar compared with the other fitting algorithms.

Figure 4 panel (**b**) shows the linear correlations for *r*-ICC across all WM voxels using a leave-one-out method (that is, correlations between “all fits” and “all fits less one fit”). The correlations confirm the ICC results. By removing CAMINO–RESTORE and AFNI–LLS, the *r* index increased.

### 3.9. Combined Results Using All Fits and All Acquisitions

**Figure 5:**
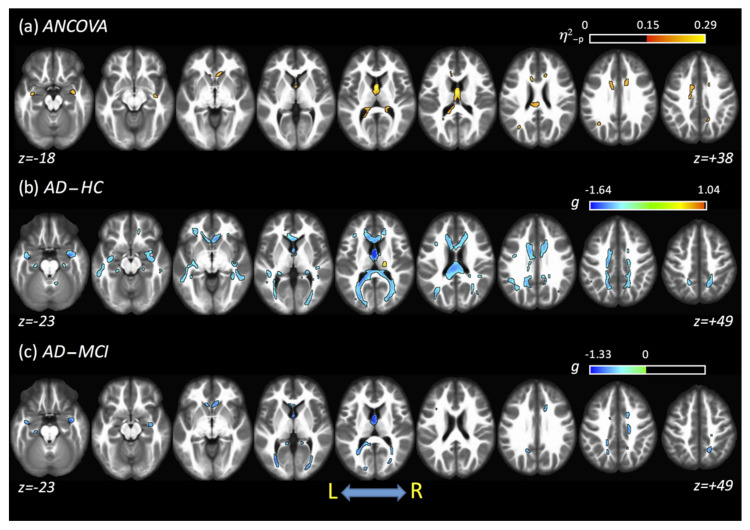
Results obtained using an average of all diffusion tensor imaging (DTI) acquisitions and fits. (**a**) ANCOVA analysis shows differences across all groups. (**b**,**c**) show the differences between AD and HC and between AD and mild cognitive impairment (MCI), respectively. No differences were found for the HC–MCI comparison.

The averaged results obtained from all fits and DTI acquisitions are shown in Figure 5. For *η*^2^_-p_, large clusters of differences across all groups were found in the fornix (87%) and CC (cluster covered around 6.4% of CC). From post hoc analysis, differences were found between AD and HC and between AD and MCI. For the AD–HC comparison, lower FA values in AD, compared with HC, were found mainly in the CCG, forceps minor and major, CC, tapetum, fornix, and posterior thalamic radiation (PTR). However, higher values of FA were found in AD compared with HC in the right CST and right posterior limb of IC. For the AD–MCI comparison, lower FA values in AD were found mainly in the forceps major, CC, fornix, right anterior corona radiata (ACR), and PTR, while no clusters of higher FA were found for AD compared to MCI. When all data (acquisitions and fits) were combined, no significant clusters were found between the HC and MCI groups. Table 3 shows a complete summary of these results.

## 4. Discussion

In this study, we analyzed WM microstructural differences using FA maps across three aging populations (HC, MCI, and AD) and compared the differences using three DTI acquisitions (from two vendors) and nine DTI fitting algorithms. Due to the different number of subjects within each group, the effect-size (reported as *η*^2^_-p_ from ANCOVA) was used to compare the magnitude of differences across acquisitions, analyses, and groups [27,28]. Through this analysis, differences across groups were observed using all acquisitions and fits. However, differences were also observed across acquisitions and fitting methods. For example, fewer significant clusters of group-wise difference were found with SI54 compared with GE48 and SI30, where the latter generally showed larger and more clusters. Within each acquisition, consistency across all fitting algorithms was high, although results of LLS–AFNI were the least similar relative to the other fitting algorithms, followed by CAMINO–RESTORE.

Distinguishing between HC and MCI has important implications for early disease detection, particularly with the development of potential disease-modifying therapies that are likely to be most effective in the early stages of the disease. In terms of the ability to distinguish between groups, it is important to note that differences between AD and MCI groups were not found with SI54 and that differences between HC and MCI were not found with SI30, suggesting that the ability to detect group-wise differences may depend on the acquisition (including the number of directions). The SI30 acquisition included only 30 diffusion-encoding directions, which is close to the lower “limit” of orientations for robust anisotropy estimation [33]. More specifically, previous simulations have demonstrated that at least 20 unique sampling orientations are needed for anisotropy estimation, and at least 30 tensor orientations are required for robust estimation of tensor orientation. Schemes with a lower number of sampling orientations may introduce bias and spurious correlations between tensor orientation and apparent diffusion characteristics. In general, a higher number of diffusion directions will yield more robust DTI-derived metrics. However, for the comparison between HC and MCI, only minimal differences between these groups were detected using the other acquisitions (GE48: left CCG; SI54: splenium of CC and fornix). However, as all three acquisitions were not acquired in the same cohort, the impact of different individual study populations cannot be discounted.

Variability related to different fitting procedures of DTI data can be traced to the underlying regression methods. The LLS regression is the most basic and generally the fastest model used in diffusion MRI for the estimation of diffusion parameters. This method incorrectly assumes that data outliers are homogeneously distributed; therefore, it does not appropriately de-weight their contributions. On the other hand, WLLS assigns a weight according to how much the original noise variation is affected by the logarithmic transform of the data. This fitting algorithm is slightly slower than LLS but is more precise. In NLLS algorithms, the estimation of the tensor is performed directly from the signal, and iterative regression is used to minimize the error between predicted and observed signal intensity. Therefore, these methods are slower and more computationally expensive compared to LLS and WLLS. LLS, WLLS, and NLLS fits only take into account the signal variability produced by thermal noise, but signal variability is also influenced by physiological noise, which varies both spatially and temporally. Physiological noise can be associated with subject motion, cardiac pulsation, respiration, and/or system instabilities. Physiological noise does not have a known parametric distribution and is usually addressed statistically by including different robust estimators, such as in the RESTORE method. In this study, we found good agreement across all fits, although both CAMINO–RESTORE and AFNI–LLS had less similarity compared with the other fits. Within one regression type (e.g., linear), differences across software tools could be attributed to different default choices of the underlying fitting algorithms, initial guesses, upper and lower bounds, and sensitivity to local minima. For higher consistency, we recommend using the default option in AFNI, which is NLLS. On the other hand, as CAMINO–RESTORE reduces the effect of physiological noise, the use of this method could improve tensor estimation, particularly in aging populations that may exhibit higher degrees of physiological noise [34].

Many studies have used DTI analysis in the assessment of AD (and in some cases MCI) cohorts [8]. However, no previous studies to our knowledge have compared results obtained from different DTI fitting procedures and from different acquisitions in the context of this disease. In healthy controls and patients with Tourette syndrome, Maximov et al. demonstrated that the agreement and reliability of TBSS results depended on the applied DTI fitting algorithm [29], while Bergamino et al. showed differences in DTI results in the analysis of depression when different DTI fits and analyses were employed [35]. Both authors recommended the analysis of DTI data using different fitting algorithms to have more robust and accurate results, in the absence of a ground-truth [36]. Correspondingly, we found robust differences across groups primarily inside the CC and the fornix when all results were combined. More specifically, differences between AD and HC were found in the CCG, forceps minor and major, CC and fornix, while differences between AD and MCI were found in the forceps major, CC, fornix, and ACR. Interestingly, no differences were found between HC and MCI using FA, consistent with other studies leveraging ADNI data [37]. However, other DTI-based biomarkers such as axial and radial diffusivities may be more sensitive to the early subtle changes associated with MCI than composite biomarkers like FA. We also found two clusters where FA in AD was higher than in HC in the right CST and in the right posterior limb of IC, which may be the result of a loss of crossing fibers due to AD-related neurodegeneration [38].

The fornix is the major output tract of the hippocampus and thus plays a critical role in memory function. Given its anatomical and functional importance, fornix pathology has been implicated in both MCI and AD [39]. In a cross-sectional study, Mielke et al. found AD patients have lower FA in the fornix than both HC and MCI [40]. Lower FA values, coupled with higher mean diffusivity, were also observed in the fornix and splenium in AD compared with MCI and HC cohorts [41]. In the present study, we found large clusters of reduced FA in the fornix for AD compared to HC (covering approximately 89% of the fornix) and MCI (covering approximately 83%). This may indicate that the fornix is a critical WM area involved in AD pathology, though definitive post-mortem studies directly comparing DTI and pathological changes remain elusive.

The role of the CC in AD is less consistent than that of the fornix. For instance, several authors did not observe any changes in DTI-related metrics in the CC in AD subjects [42,43,44], while other studies have shown reduced FA in AD subjects in the posterior regions of the CC [45,46] or in the anterior region of the CC [45,47]. Additionally, Xie et al. found lower FA in the genu and anterior body of the CC [48], while Preti et al. found differences in the CC between AD and both HC and MCI cohorts [49]. Combining all fits and all acquisitions, we found lower FA values in AD compared to HC in the genu, body, and splenium of the CC (all clusters covered about 38% of the CC) and similar but smaller clusters for the AD–MCI comparison (about 5% of the CC). This may mean that WM microstructural changes in the CC occur later in the pathological cascade.

There are several limitations in this study. First, we only used single-shell DTI acquisitions available from ADNI, and thus, we were not able to evaluate different fitting algorithms with multi-shell data, which may yield improved microstructural metrics. While ADNI is continuously expanding, at the time of data analysis, only one multi-shell acquisition was available (with 126 directions), and with very few AD subjects. Second, the data available in ADNI for this study were not matched across groups and acquisitions; for example, there were fewer AD subjects relative to HC and MCI, especially for SI54. As a primary goal of ADNI is to develop biomarkers for the earliest phases of AD, higher numbers of HC and MCI can be expected. ANCOVA may not be appropriate when variances are heterogeneous across groups, particularly in the presence of unbalanced sample sizes; however, when sample sizes are unequal and variance is not heterogeneous, as verified in the present study, ANCOVA remains the recommended test [26]. Additionally, to account for unequal sample sizes across acquisitions, we provide the main results as effect sizes, which reflect the magnitude of the differences. Finally, this study only analyzed FA, which is the most commonly used DTI index [15]. Other DTI related metrics, such as radial, axial, and mean diffusivity, may also be of interest, and work is ongoing to assess the impact of different fitting methods on those parameters.

In conclusion, we found differences in the FA maps derived from different dMRI acquisitions and from different DTI fitting methods in the analysis of AD and MCI subjects, compared to HC. These differences were more consistent across fits as the number of directions increased, suggesting that aging studies could be improved with more dMRI directions and that acquisitions with less than 30 directions should be avoided. In terms of the fits, we found that AFNI–LLS had the least similarity compared with the other algorithms, while all other fitting algorithms produced highly similar results, suggesting flexibility for end-users to choose between LLS, WLLS, and NLLS. CAMINO–RESTORE also produced less similar results, but this may be due to compensation for physiological effects. By combining all acquisitions and all fits, we observed differences between AD and HC and between AD and MCI, particularly in the fornix and CC, suggesting robust changes in FA in these regions in AD. Researchers should be aware that potential differences in the results related to the choice of DTI fit type may have implications for comparisons across studies with varying methodologies, particularly in the context of subtle WM changes associated with early AD pathology. Overall, these results show that identifying the most robust DTI analysis methods, including the choice of fitting algorithm and software tool, is a critical step to provide more reliable DTI-based neuroimaging biomarkers for assessing microstructural changes in AD.

## Figures and Tables

**Table 1:** Subject characteristics and clinical scores.

**Across Groups**					**Dunn’s Test of Multiple Comparisons with Holm–Sidak Adjustment Z; *p*-Value**		
	**AD**	**HC**	**MCI**	**KRUSKAL–WALLIS RANK SUM TEST**	***AD* vs *HC***	***AD* vs. *MCI***	***HC* vs. *MCI***
**SI30**							
N (female)	7 (3)	38 (25)	23 (14)	–	–	–	–
AGE	75.9 (8.6)	70.1 (4.0)	70.9 (8.1)	H = 5.8; *p* = 0.054	–	–	–
MMSE	19.6 (4.9)	29.4 (0.9)	28.3 (1.7)	H = 26.6; *p* < 0.001 *	Z = −4.96; *p* < 0.001 *	Z = −3.16; *p* = 0.002 *	Z = 2.56; *p* = 0.010 *
MoCA	12.6 (4.1)	25.7 (2.6)	22.5 (3.8)	H = 23.9; *p* < 0.001 *	Z = −4.64; *p* < 0.001 *	Z = −2.48; *p* = 0.013 *	Z = 2.55; *p* = 0.011 *
ADAS	23.1 (4.0)	8.6 (2.8)	11.7 (3.0)	H = 26.8; *p* < 0.001 *	Z = 4.84; *p* < 0.001 *	Z = 2.54; *p* = 0.011 *	Z = −2.92; *p* = 0.003 *
**GE48**							
N (female)	12 (2)	41 (27)	20 (9)	–	–	–	–
AGE	75.6 (5.4)	70.8 (5.8)	72.7 (9.3)	H = 4.7; *p* = 0.094	–	–	–
MMSE	23.7 (1.9)	29.3 (0.9)	27.3 (2.0)	H = 40.6; *p* < 0.001 *	Z = −5.78; *p* < 0.001 *	Z = −2.64; *p* = 0.008 *	Z = 3.42; *p* = 0.001 *
MoCA	16.1 (3.3)	25.4 (2.6)	21.4 (3.4)	H = 35.7; *p* < 0.001 *	Z = −5.59; *p* < 0.001 *	Z = −2.09; *p* = 0.036 *	Z = 3.52; *p* < 0.001 *
ADAS	22.2 (6.2)	8.5 (2.3)	14.6 (5.2)	H = 38.0; *p* < 0.001 *	Z = 5.68; *p* < 0.001 *	Z = 1.93; *p* = 0.054	Z = −3.85; *p* < 0.001 *
**SI54**							
N (female)	11 (3)	80 (56)	36 (18)	–	–	–	–
AGE	72.1 (9.2)	69.6 (6.2)	71.7 (6.5)	H = 6.13; *p* = 0.051	–	–	–
MMSE	24.1 (3.2)	28.9 (1.2)	27.3 (2.3)	H = 35.76; *p* < 0.001*	Z = −5.09; *p* < 0.001 *	Z = −2.39; *p* = 0.02 *	Z = 4.06; *p* < 0.001 *
MoCA	17.6 (3.0)	25.3 (2.7)	21.6 (2.8)	H = 45.41; *p* < 0.001*	Z = −5.65; *p* < 0.001 *	Z = −2.20; *p* = 0.03 *	Z = 4.76; *p* < 0.001 *
ADAS	19.4 (4.6)	8.5 (2.2)	12.6 (3.8)	H = 47.34; *p* < 0.001*	Z = 5.93; *p* < 0.001 *	Z = 2.54; *p* = 0.01 *	Z = −4.63; *p* < 0.001 *
**Across Scanners**					**Dunn’s Test of Multiple Comparisons with Holm-Sidak Adjustment Z; *p*-Value**		
	**SI30**	**GE48**	**SI54**	**KRUSKAL–WALLIS RANK SUM TEST**	***GE48* vs. *SI30***	***GE48* vs. *SI54***	***S30* vs. *SI54***
**HC**							
N (female)	38 (25)	41 (27)	80 (56)	–	–	–	–
AGE	70.08 (4.04)	70.79 (5.75)	69.65 (6.27)	H = 1.50; *p* = 0.52		–	–
MMSE	29.37 (0.94)	29.33 (0.89)	28.96 (1.16)	H = 3.91; *p* = 0.14	–	–	–
MoCA	25.71 (2.61)	25.45 (2.63)	25.27 (2.68)	H = 0.39; *p* = 0.82	–	–	–
ADAS	8.59 (2.76)	8.47 (2.25)	8.54 (2.16)	H = 0.10; *p* = 0.95	–	–	–
**MCI**							
N (female)	23 (14)	20 (9)	36 (18)	–	–	–	–
AGE	70.89 (8.14)	72.68 (9.31)	71.75 (6.56)	H = 0.21; *p* = 0.90	–	–	–
MMSE	28.30 (1.69)	27.30 (2.00)	27.30 (2.25)	H = 4.41; *p* = 0.11	–	–	–
MoCA	22.47 (3.81)	21.44 (3.37)	21.62 (2.82)	H = 1.59; *p* = 0.45	–	–	–
ADAS	11.74 (3.02)	14.58 (5.18)	12.55 (3.77)	H = 2.38; *p* = 0.30	–	–	–
**AD**							
N (female)	7 (3)	12 (2)	11 (3)	–	–	–	–
AGE	75.8 (8.6)	75.6 (5.4)	72.1 (9.2)	H = 1.14; *p* = 0.57	–	–	–
MMSE	19.52 (4.91)	23.62 (1.92)	24.14 (3.21)	H = 5.97; *p* = 0.05	–	–	–
MoCA	12.57 (4.12)	16.11 (3.43)	17.51 (3.03)	H = 6.90; *p* = 0.03 *	Z = 1.55; *p* = 0.122	Z = −1.28; *p* = 0.201	Z = −2.62; *p* = 0.010 *
ADAS	23.11 (4.02)	22.23 (6.71)	19.42 (4.61)	H = 4.62; *p* = 0.10	–	–	–

Group demographics for each acquisition with the Mini-Mental State Examination (MMSE), Montreal Cognitive Assessment (MoCA), and Alzheimer’s Disease Assessment Scale (ADAS) scores, given as mean (SD). * Significant differences across groups and acquisitions.

**Table 2:** Fitting algorithms and software tools used in this study.

Fit Algorithm	Tool	Web Page	Command	Option
LLS	FSL	https://fsl.fmrib.ox.ac.uk/fsl/fslwiki	dtifit	default
LLS	DSI Studio	http://dsi-studio.labsolver.org/	dsi_studio	default
LLS	CAMINO	http://camino.cs.ucl.ac.uk/index.php	modelfit	-inversion 1 (ldt)
LLS	AFNI	https://afni.nimh.nih.gov/	3dDWItoDT	-linear
WLLS	FSL	https://fsl.fmrib.ox.ac.uk/fsl/fslwiki	dtifit	--wls
WLLS	CAMINO	http://camino.cs.ucl.ac.uk/index.php	modelfit	-inversion 7 (ldt_wtd)
NLLS	CAMINO	http://camino.cs.ucl.ac.uk/index.php	modelfit	-inversion 4 (nldt_pos nldt)
NLLS	AFNI	https://afni.nimh.nih.gov/	3dDWItoDT	-nonlinear
RESTORE	CAMINO	http://camino.cs.ucl.ac.uk/index.php	restore	default

Fitting algorithms and software tools used in this study. The default for AFNI is –*nonlinear*.

**Table 3:** Summary of results for FA by combining all fits and all acquisitions.

	**ANCOVA**	**AD vs. HC**		**AD vs. MCI**
**JHU WM Tractography**	**Volume %**	**AD < HC Volume %**	**AD > HC Volume %**	**AD < MCI Volume %**
ATR	0.67	1.43	–	0.23
Right CST	–	–	0.34	0.68
CCG	0.89	11.11	–	0.97
Cingulum Hippo	–	5.13	–	3.30
Forceps Major	1.57	14.05	–	8.27
Forceps Minor	1.05	7.13	–	1.52
Left IFOF	–	5.51	–	2.14
Right IFOF	0.36	6.74	–	2.49
ILF	0.14	6.11	–	2.67
UF	0.86	7.74	–	3.40
**ICBM-DTI-81 WM**	**Volume %**	**AD < HC Volume %**	**AD > HC Volume %**	**AD < MCI Volume %**
Genu of CC	6.05	42.04	–	10.52
Body of CC	9.59	44.06	–	2.30
Splenium of CC	3.60	25.97	–	2.27
Fornix	87.38	89.05	–	83.11
Right posterior limb of IC		–	1.77	–
Left retrolenticular IC	–	4.71	–	–
Right ACR	–	5.15	–	0.74
Right ACR	0.39	3.85	–	7.49
Left ACR	0.68	4.04	–	–
Right PCR	–	7.76	–	–
Left PCR	–	7.48	–	1.95
PTR	–	14.70	–	7.57
SS	0.34	4.48	–	–
Right EC	0.77	2.82	–	0.62
Left EC	–	5.29	–	–
Fornix (cres)/Stria terminalis	8.34	17.78	–	7.03
Right tapetum	–	24.75	–	4.11
Left tapetum	1.22	8.90	–	2.56

Summary of results for FA by combining all fits and all acquisitions. The ‘Volume %’ is the percent volume of the clusters within the corresponding brain area. No significant differences were observed between HC and MCI groups during post hoc comparisons.

## Data Availability

All data used in this study can be obtained from the ADNI database (https://ida.loni.usc.edu/).

## Supplementary Materials

The following are available online at https://www.mdpi.com/2379-1381/7/1/3/s1, Figure S1: AD vs. HC post-hoc comparison for all fitting algorithms for cluster volumes and *g* index with the relative pairs linear fits correlations for (a) SI30, (b) GE48, and (c) SI54. Significant clusters with |*g*| > 0.85 and size > 100 voxels. Figure S2: AD vs. MCI post-hoc comparison for all fitting algorithms for cluster volumes and *g* index with the relative pairs linear fits correlations for (a) SI30, (b) GE48. No differences between these two groups were detected by SI54. Significant clusters with |*g*| > 0.85 and size > 100 voxels. Figure S3: MCI vs HC post-hoc comparison for all fitting algorithms for cluster volumes and *g* index with the relative pairs linear fits correlations for (a) GE48 and (b) SI54. No differences between these two groups were detected by SI30. Significant clusters with |*g*| > 0.85 and size > 100 voxels. Figure S4: Voxel-based Spearman’s correlations between cognitive scores (MoCA, MMSE, and ADAS) and the FA values from all DTI fits and acquisitions. Significant clusters with |*r*| > 0.50 and size > 100. No correlations were found for Siemens 54, Table S1: Abbreviations for the WM areas used in this study.

## Abbreviations

Alzheimer’s disease (AD), mild cognitive impairment (MCI), positron emission tomography (PET), magnetic resonance imaging (MRI), diffusion tensor imaging (DTI), white matter (WM), fractional anisotropy (FA), axial diffusivity (AxD), radial diffusivities (RxD), corpus callosum (CC), uncinate fasciculus (UF), tract-based spatial statistics (TBSS), linear least squares (LLS), weighted linear least squares (WLLS), non-linear least squares regression (NLLS), robust estimation of tensors by outlier rejection (RESTORE), informed RESTORE (iRESTORE), healthy control (HC), Alzheimer’s Disease Neuroimaging Initiative (ADNI), General Electric (GE), Montreal Cognitive Assessment (MoCA), Mini-Mental State Examination (MMSE), Alzheimer’s Disease Assessment Scale (ADAS), intraclass correlation coefficient (ICC), functional magnetic resonance imaging of the brain (FMRIB), advanced normalization tools (ANTs), symmetric image normalization (SyN), standard deviation (SD), cingulate gyrus (CCG), superior longitudinal fasciculus (SLF), inferior cerebellar peduncle (ICP), cerebral peduncle (CP), cortical spinal tract (CST), posterior thalamic radiation (PTR), anterior corona radiata (ACR), Internal capsule (IC).
