# Supplementary material for: Systematic Assessment of the Impact of DTI Methodology on Fractional Anisotropy Measures in Alzheimer’s Disease
Source: Tomography. 2021 Feb 6;7(1):20–38. doi: 10.3390/tomography7010003 (PMC7934686; doi:10.3390/tomography7010003)
Supplement: Supplementary file 1 [file tomography-07-00003-s001.pdf]

Supplementary Table S1: Abbreviations for the WM areas used in this study.

Name WM area	Abbreviation
Anterior thalamic radiation	ATR
Cortical spinal tract	CST
Cingulum cingulate gyrus	CCG
Cingulum Hippo	cingulum Hippo
Forceps Major	forceps major
Forceps Minor	forceps minor
Inferior fronto-occipital fasc	IFOF
Inferior longitudinal fasc	ILF
Superior longitudinal fasc	SLF
Uncinate fasc	UF
Middle cerebellar peduncle	MCP
Corpus callosum	CC
Fornix	fornix
Medial lemniscus	medial lemniscus
Inferior cerebellar peduncle	ICP
Superior cerebellar peduncle	SCP
Cerebral peduncle	CP
Internal capsule	IC
Anterior corona radiata	ACR
Superior corona radiata	SCR
Posterior corona radiata	PCR
Posterior thalamic radiation	PTR
Sagittal stratum	SS
External capsule	EC
Superior fronto-occipital fasciculus	SFOC

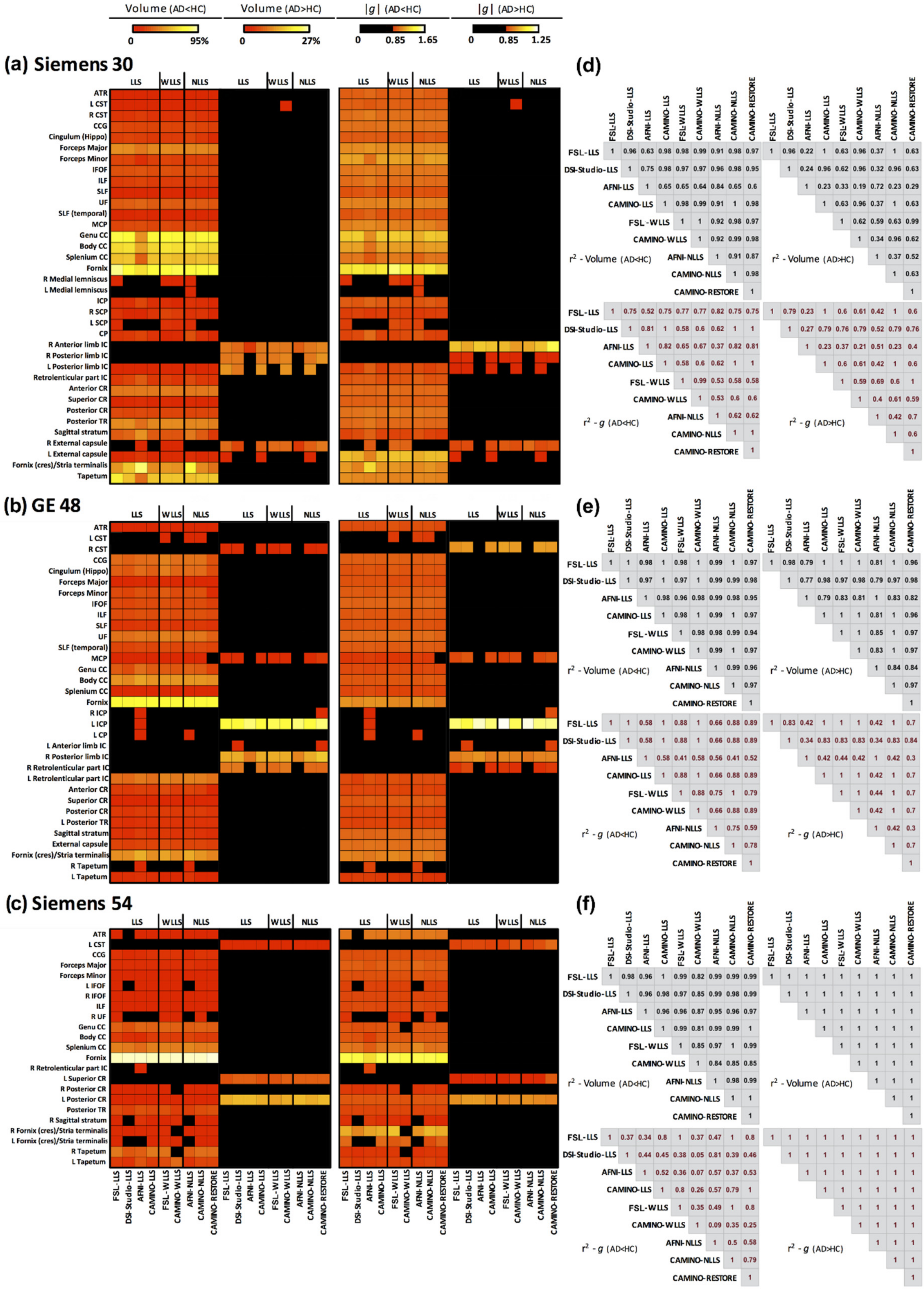

Supplementary figure S1

AD *vs* HC post-hoc comparison for all fitting algorithms for cluster volumes and *g* index with the relative pairs linear fits correlations for (a) SI30, (b) GE48, and (c) SI54. Significant clusters with |*g*| > 0.85 and size > 100 voxels.

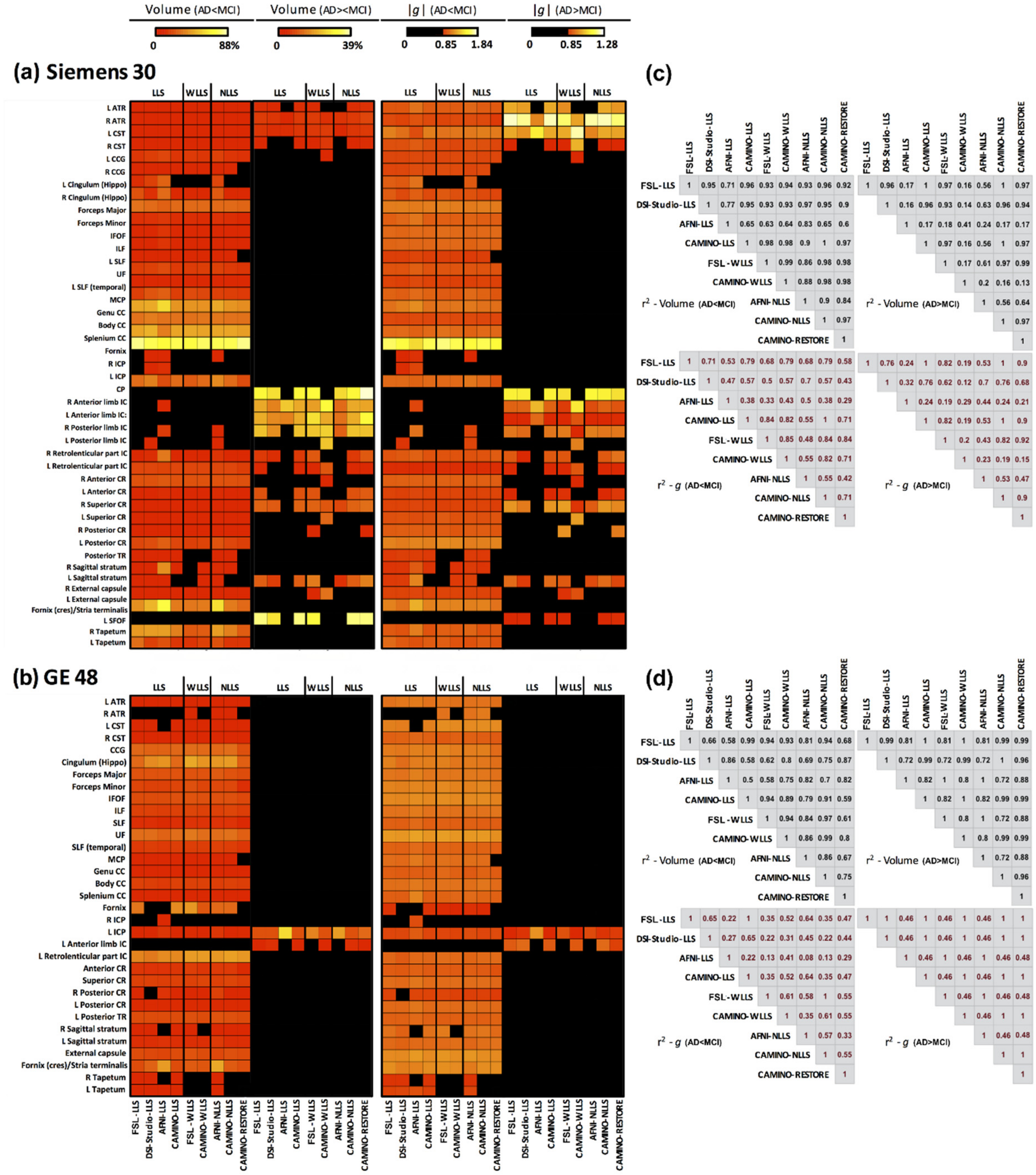

Supplementary figure S2

AD *vs* MCI post-hoc comparison for all fitting algorithms for cluster volumes and g index with the relative pairs linear fits correlations for (a) SI30, (b) GE48. No differences between these two groups were detected by SI54. Significant clusters with $|g| > 0.85$ and size > 100 voxels.

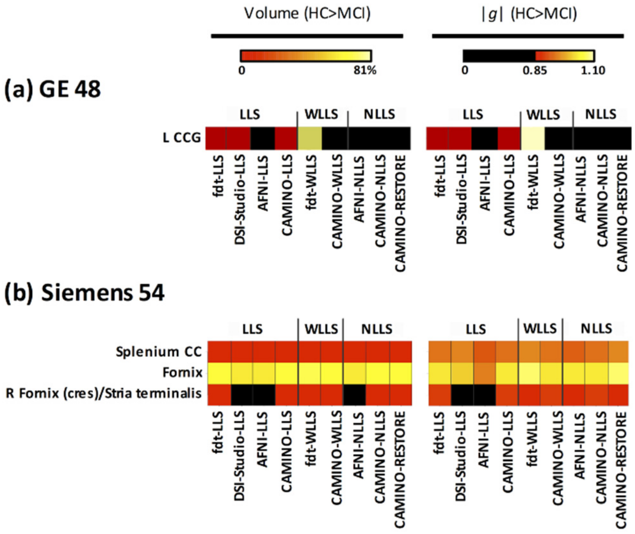

Supplementary figure S3

MCI *vs* HC post-hoc comparison for all fitting algorithms for cluster volumes and g index with the relative pairs linear fits correlations for (a) GE48 and (b) SI54. No differences between these two groups were detected by SI30. Significant clusters with $|g| > 0.85$ and size > 100 voxels.

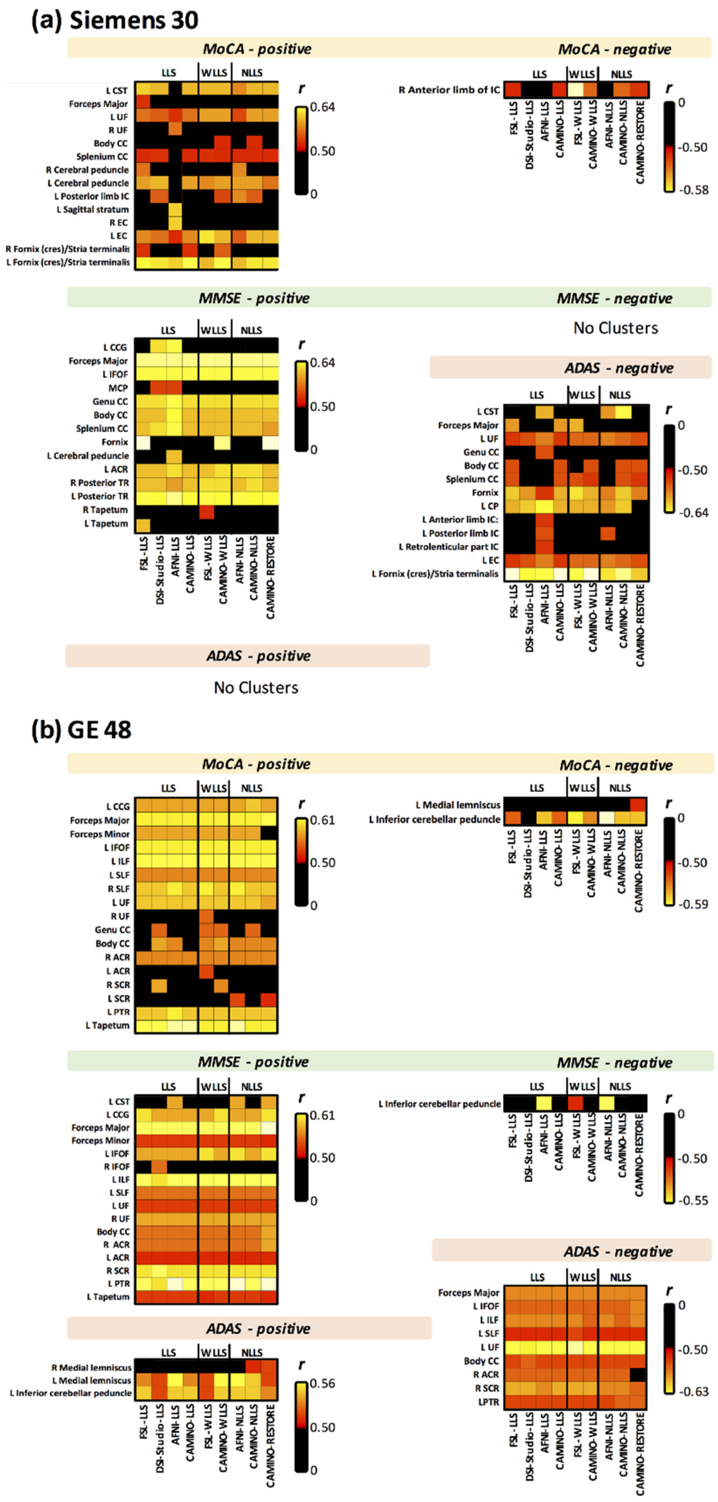

Supplementary figure S4

Voxel-based Spearman's correlations between cognitive scores (MoCA, MMSE, and ADAS) and the FA values from all DTI fits and acquisitions. Significant clusters with |*r*| > 0.50 and size > 100. No correlations were found for Siemens 54.
